# A Retrospective Study of Routine Preoperative Blood Grouping and Saving in Laparoscopic Surgeries: A Minimally Utilized Expenditure

**DOI:** 10.7759/cureus.68557

**Published:** 2024-09-03

**Authors:** Sadhasivam Ramasamy, Seshu Kumar Bylapudi, Sidharth Kumar, Jatinder Singh, Rafique Umer Harvitkar, Giri Babu Gattupalli, Yousaf Tanveer, Sara Harvitkar, Moustafa Mohmoud, Mohamadali Sayyad

**Affiliations:** 1 General Surgery, Queens Hospital Burton, Burton, GBR; 2 General Surgery, Kettering General Hospital, Kettering, GBR; 3 General Surgery, Sandwell and West Birmingham Hospitals National Health Service (NHS) Trust, Sandwell, GBR; 4 Hepato-Pancreato-Biliary Unit, Leicester Glenfield Hospital, Leicester, GBR; 5 General Surgery, Queen Alexandra University Hospital, Portsmouth, GBR; 6 Surgery, Diana Princess of Wales Hospital, Grimsby, GBR; 7 General Surgery, Craigavon Area Hospital, Craigavon, GBR; 8 Psychology, Southern Health National Health Service (NHS) Foundation Trust, Havant, GBR; 9 Orthopedic Surgery, Queen Alexandra University Hospital, Portsmouth, GBR; 10 Urology, Ishaan Urology and Kidney Care Hospital, Mumbai, IND

**Keywords:** laparoscopy, laparoscopic cholecystectomy, blood transfusion, blood group and save, laparoscopic appendicectomy

## Abstract

Introduction

Patients scheduled for laparoscopic cholecystectomy and laparoscopic appendicectomy typically undergo routine preoperative blood grouping and saving (G&S). Despite the low incidence of blood transfusion in this context, the acquisition and processing of G&S samples incur a cost of £31 ($40) per sample. This study aims to review blood transfusion usage in these procedures to determine whether routine G&S sampling is clinically necessary or represents an avoidable expense.

Methods

A retrospective case note analysis was conducted on patients who underwent laparoscopic cholecystectomy and laparoscopic appendicectomy from January 2019 to June 2020. Collected data included the timing of G&S, preoperative and postoperative hemoglobin levels, timing of blood transfusions, and the number of units transfused.

Results

Six hundred and thirteen patients were involved in the study. Among the 323 patients who had laparoscopic cholecystectomy, 256 (78.8%) underwent preoperative G&S sampling. Of the 290 patients who had laparoscopic appendicectomy, 190 (65.5%) received preoperative G&S sampling. Notably, none of the 613 patients required a blood transfusion within 30 days of their surgery. The total cost of G&S for the cohort amounted to £22,196 ($28,425).

Conclusions

The findings suggest that routine G&S sampling is an unnecessary expenditure for patients undergoing elective laparoscopic appendicectomy or cholecystectomy. It is recommended that G&S sampling be reserved for high-risk groups to optimize resource allocation and reduce unnecessary costs.

## Introduction

In the United Kingdom, approximately 69,000 laparoscopic cholecystectomies and 44,000 laparoscopic appendicectomies are performed annually, making these two of the most commonly conducted basic laparoscopic procedures in general surgery [1]. Laparoscopic cholecystectomy is the gold standard treatment for most gallbladder pathologies, including gallstones and gallbladder polyps. Similarly, laparoscopic appendicectomy has gained significant popularity over the past two decades for treating appendicitis, surpassing open techniques [2-4].

The most common sources of bleeding in laparoscopic cholecystectomy are the cystic artery and the gallbladder bed, both of which are typically managed laparoscopically. During laparoscopic appendicectomy, bleeding can occur from the appendicular artery within the mesoappendix, which can usually be controlled laparoscopically. Uncontrollable bleeding from retroperitoneal vessels during laparoscopic appendicectomy is rare. Additionally, major vascular injuries can occur, though infrequently (0.07-0.11%), during the creation of pneumoperitoneum [5-7].

Advancements in laparoscopic instrumentation, techniques, and training have made basic laparoscopic operations safer, with minimal blood loss during surgery. Consequently, the need for blood transfusions has become exceedingly rare, with current intraoperative and postoperative transfusion rates for laparoscopic cholecystectomy ranging from 0% to 1.4%[7-10].

ABO and rhesus antigen testing are used to determine a patient's blood group, while saving the sample allows for cross-matching, which involves checking for antibodies prior to a blood transfusion. This practice, historically known as "group and save" (G&S), was routinely performed as part of the preoperative workup during the era of open surgery. Despite the very low rate of intraoperative and postoperative blood transfusions, G&S is still routinely performed preoperatively before most abdominal operations, including laparoscopic cholecystectomy and laparoscopic appendicectomy [9-11].

This study aimed to examine the frequency of perioperative blood transfusions and perform a cost analysis of G&S samples collected prior to laparoscopic cholecystectomy and appendicectomies.

## Materials and methods

This retrospective cross-sectional study was conducted at Milton Keynes University Hospital in the United Kingdom. The study aimed to evaluate the use of G&S blood samples in patients undergoing laparoscopic cholecystectomy or laparoscopic appendicectomy. Patients who underwent these procedures between January 1, 2019, and June 30, 2020, were identified using the Office of Population Censuses and Surveys coding from the hospital's information department. The study received approval from the hospital's clinical governance department, and written informed consent was waived due to the study's design as a quality improvement audit.

All laparoscopic appendicectomies were performed as emergency operations. Two hundred and six (63.8%) had emergency cholecystectomies. One hundred seventeen (36.2%) had elective cholecystectomies. Patients were identified through the hospital’s electronic theater database. Each patient's records were meticulously reviewed using the hospital's eCARE software (CernerWorks) to determine the number and timing of G&S blood samples sent and any blood product transfusions administered. This review process included examining patient demographics, comorbidities, and anticoagulant usage to provide a comprehensive overview of the patient population and their clinical needs.

All patients who met the criteria were included in the analysis, with no exclusions, ensuring a thorough and unbiased assessment of the data. The comprehensive review of patient records allowed for an accurate determination of the use and necessity of G&S blood samples in these surgical procedures.

To assess the financial and temporal impact of the G&S process, the study also gathered data on the cost and approximate time involved from the hospital's transfusion department. This information was crucial in evaluating the efficiency and resource utilization associated with G&S blood samples in the context of laparoscopic surgeries.

Overall, the study aimed to provide valuable insights into the practices surrounding G&S blood sampling in laparoscopic surgeries, with the ultimate goal of informing and improving clinical guidelines and resource management within the hospital.

## Results

Patient demographics

During the study period, 290 patients underwent laparoscopic appendicectomies, and 323 patients had laparoscopic cholecystectomy. The patient demographics are summarized in Table 1 below.

**Table 1:** Patient demographics ASA: American Society of Anaesthesiologists

	Laparoscopic cholecystectomy (n=323)	Laparoscopic appendicectomy (n=290)
Mean age	50.1 ± 14.67	31 ± 17.21
Sex ratio (F:M)	1:0.9	2.4:1
ASA score		
1	70 (21.67%)	153 (52.7%)
2	228 (70.5%)	128 (44.1%)
3	24 (7.4%)	9 (3.1%)
4	1 (0.3%)	0 (0%)
Anticoagulant use	14 (4%)	8 (2.7%)

The study on laparoscopic appendicectomy involving 290 patients revealed a mean age of 31.02 years, with a female-to-male ratio of 1:0.9. Among the patients, 190 (65.5%) had one preoperative G&S sample, 44 (15.1%) had two G&S samples, and five patients (2%) required cross-matching. All laparoscopic appendicectomies were performed as emergency operations. None of the patients required intraoperative or immediate postoperative blood transfusions (Table 2).

**Table 2:** Preoperative G&S and transfusion rate G&S: group and save

Procedure	n	1 G&S (%)	2 G&S (%)	Cross-match (%)	Transfusion (%)
Laparoscopic appendicectomy	290	190 (65.5%)	44 (15.1%)	5 (2%)	0
Laparoscopic cholecystectomy	323	236 (73%)	48(14.8%)	13 (4%)	0

**Figure 1:**
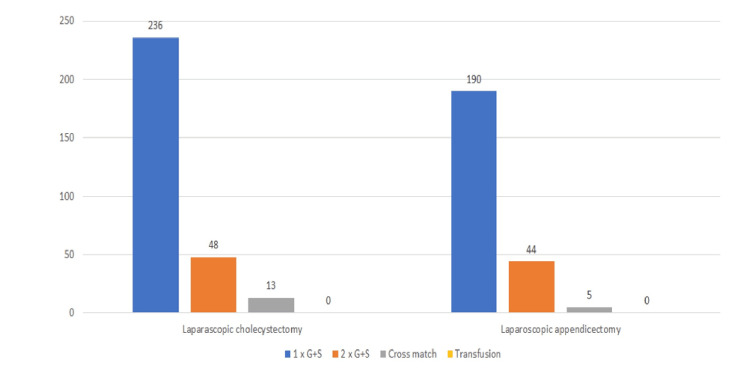
Number of G&S and cross-match and transfusion rate G&S: group and save

A study on laparoscopic cholecystectomy revealed that the mean age of the patients was 50.11 years, with a female-to-male ratio of 2.4:1. Among the 323 patients included, 206 (63.8%) underwent emergency cholecystectomies while 117 (36.2%) had elective procedures. In terms of preoperative blood sampling, 236 patients (73%) had one group and save (G&S) sample, 48 patients (14.8%) had two G&S samples, and 13 patients (4%) required cross-matching. No patients required blood transfusions either intraoperatively or immediately postoperatively (Figure 1).

Blood transfusion rate

The rate of intraoperative or immediate postoperative transfusion was zero (0%) for the entire patient cohort, including both laparoscopic appendicectomy and cholecystectomy, over the 18-month study period.

Cost analysis

The cost for one G&S sample was £31 ($40) at our trust, including value-added tax, and it took up to 40 minutes to process the sample. The total cost for G&S samples over the study period was £18,910 ($24,831), excluding the consumables associated with blood sampling.

## Discussion

Our study underscores the negligible perioperative need for blood transfusion in laparoscopic appendicectomy and cholecystectomy, aligning with findings from existing literature [5]. Despite this well-documented low transfusion risk, routine G&S sampling before these procedures remains common practice across hospitals in the United Kingdom. Each hospital adheres to its own Maximum Surgical Blood Ordering Schedule (MSBOS), which guides the necessity of G&S based on patient factors and procedural complexity. At Milton Keynes University Hospital, our MSBOS recommends one G&S before laparoscopic cholecystectomy and advises against G&S sampling prior to laparoscopic appendicectomy.

In our study, anemia was defined based on hemoglobin levels: males with hemoglobin <130 g/L and females with hemoglobin <120 g/L. Among the 290 patients who underwent appendicectomy, 12 (4.1%) were identified as anemic. Of the 323 patients who underwent cholecystectomy, 22 (6.8%) had preoperative anemia, with an observation that only two anemic cases were in the elective cholecystectomy group.

Numerous studies in the current literature advocate for safely omitting routine G&S sampling in basic laparoscopic procedures [5,11-13]. Quinn et al. specifically recommend a targeted approach, reserving G&S for patients with multiple co-morbidities or those on anticoagulation [11].

Historically, during the initial adoption of laparoscopic techniques in the 1980s and 1990s, limited technical proficiency resulted in higher complication rates, including bleeding necessitating transfusion. Consequently, routine preoperative G&S became standard practice. However, over the past four decades, laparoscopic procedures have evolved into a standard surgical approach. Surgical trainees now acquire advanced laparoscopic skills earlier in their careers, contributing to reduced bleeding risks and transfusion needs. Techniques such as open port placement have mitigated risks associated with major vascular injuries during pneumoperitoneum development [14,15].

In the rare event of a major vascular injury necessitating immediate transfusion, our hospital, like all NHS hospitals, adheres to the Major Haemorrhage Protocol. This protocol ensures immediate access to type O rhesus-negative blood stored in theaters, bypassing the need for cross-matched blood in critical situations.

Our cost analysis revealed that Milton Keynes University Hospital could have saved £18,910 ($24,838) over the 18-month study period by refraining from routine G&S sampling for laparoscopic appendicectomy and cholecystectomy patients, given the absence of any transfusion requirements during this period. This includes patients with multiple medical comorbidities, those on anticoagulation therapy, and those admitted for intraoperative concerns. In patients with preoperative anemia, these strategies may include preoperative optimization of hemoglobin levels when possible, careful intraoperative hemostasis, and postoperative monitoring to ensure patient safety and improve surgical outcomes.

While acknowledging the limitations of our retrospective, single-center study, our findings align with existing literature and support reconsideration of routine G&S sampling practices before laparoscopic appendicectomy and cholecystectomy. Moving forward, adopting a selective approach to G&S sampling could optimize resource allocation without compromising patient safety.

## Conclusions

Our study and a comprehensive literature review show that blood transfusion during or immediately after laparoscopic cholecystectomy or appendicectomy is very rare, suggesting that routine preoperative G&S sampling for these procedures is unnecessary. This change could reduce healthcare costs without compromising patient care or safety.

Adopting a selective approach to G&S sampling based on individual risk factors, such as medical comorbidities and anticoagulant use, may optimize resource utilization while maintaining clinical standards. This targeted approach promotes efficient healthcare delivery in laparoscopic surgery and could lead to significant cost savings and improved patient outcomes.
